# JAK–STAT signalling shapes the NF‐κB response in CLL towards venetoclax sensitivity or resistance via Bcl‐XL

**DOI:** 10.1002/1878-0261.13364

**Published:** 2023-02-13

**Authors:** Marco V. Haselager, Rachel Thijssen, Danique Bax, Demi Both, Francien De Boer, Simon Mackay, Julie Dubois, Clemens Mellink, Arnon P. Kater, Eric Eldering

**Affiliations:** ^1^ Department of Experimental Immunology Amsterdam UMC location University of Amsterdam The Netherlands; ^2^ Amsterdam institute for Infection & Immunity The Netherlands; ^3^ Cancer Immunology Cancer Center Amsterdam The Netherlands; ^4^ Lymphoma and Myeloma Center Amsterdam LYMMCARE Amsterdam The Netherlands; ^5^ Department of Hematology Amsterdam UMC location University of Amsterdam The Netherlands; ^6^ Ikazia Ziekenhuis Rotterdam The Netherlands; ^7^ Strathclyde Institute of Pharmacy and Biomedical Sciences University of Strathclyde Glasgow UK; ^8^ Department of Clinical Genetics Amsterdam UMC location University of Amsterdam The Netherlands

**Keywords:** chronic lymphocytic leukaemia, drug resistance, microenvironment, signalling

## Abstract

Preventing or overcoming resistance to the Bcl‐2 inhibitor venetoclax is an emerging unmet clinical need in patients with chronic lymphocytic leukaemia (CLL). The upregulation of anti‐apoptotic Bcl‐2 members through signalling pathways within the tumor microenvironment appears as a major factor leading to resistance to venetoclax. Previously, we reported that T cells can drive resistance through CD40 and non‐canonical NF‐κB activation and subsequent Bcl‐XL induction. Moreover, the T cell‐derived cytokines IL‐21 and IL‐4 differentially affect Bcl‐XL expression and sensitivity to venetoclax via unknown mechanisms. Here, we mechanistically dissected how Bcl‐XL is regulated in the context of JAK–STAT signalling in primary CLL. First, we demonstrated a clear antagonistic role of IL‐21/STAT3 signalling in the NF‐κB‐mediated expression of Bcl‐XL, whereas IL‐4/STAT6 further promoted the expression of Bcl‐XL. In comparison, Bfl‐1, another NF‐κB target, was not differentially affected by either cytokine. Second, STAT3 and STAT6 affected Bcl‐XL transcription by binding to its promoter without disrupting the DNA‐binding activity of NF‐κB. Third, *in situ* proximity ligation assays (isPLAs) indicated crosstalk between JAK–STAT signalling and NF‐κB, in which STAT3 inhibited canonical NF‐κB by accelerating nuclear export, and STAT6 promoted non‐canonical NF‐κB. Finally, NF‐κB inducing kinase (NIK) inhibition interrupted the NF‐κB/STAT crosstalk and resensitized CLL cells to venetoclax. In conclusion, we uncovered distinct crosstalk mechanisms that shape the NF‐κB response in CLL towards venetoclax sensitivity or resistance via Bcl‐XL, thereby revealing new potential therapeutic targets.

## Graphical abstract

CD40 stimulation of CLL cells leads to venetoclax resistance due to Bcl‐XL upregulation mediated by NF‐κB signalling. The T cell‐derived cytokines IL‐21 and IL‐4 differentially affect Bcl‐XL expression and sensitivity to venetoclax. Here, we uncovered distinct crosstalk mechanisms that shape the NF‐κB response in CLL towards venetoclax sensitivity or resistance via Bcl‐XL, thereby revealing new potential therapeutic targets.

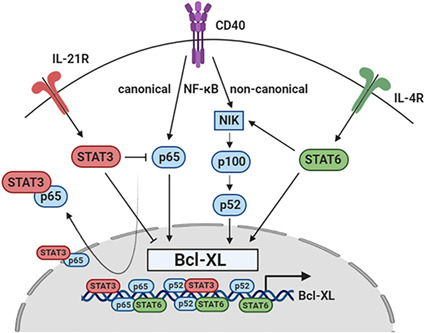

**Abbreviations**
CLL: chronic lymphocytic leukaemia
isPLA: *in situ* proximity ligation assay
JAK: Janus kinase
LN: lymph node
NIK: NF‐κB‐inducing kinase
RT‐MLPA: reverse transcription‐multiplex ligation‐dependent probe amplification assay
STAT: signal transducers and activators of transcription
STAT3C: constitutively active STAT3 mutant

## 1 Introduction

Venetoclax has been approved for previously treated chronic lymphocytic leukaemia (CLL) in 2018 and for previously untreated CLL in 2019 [1, 2, 3]. Although this has led to a breakthrough in the treatment of CLL, resistance to venetoclax is inevitable. Acquired BCL2 mutations have been identified as a potential resistance mechanism, but only a small number of cases show the presence of mutations, and only after prolonged treatment duration [4, 5], suggesting that additional mechanisms play a role. CLL is a malignancy that is highly dependent on interactions with the microenvironment. In the lymph node (LN), CD40L‐presenting follicular T cells may promote microenvironment‐induced drug resistance by activating CD40 in CLL cells and the secretion of cytokines that affect drug resistance [6, 7, 8, 9, 10]. We and others have demonstrated that CD40 stimulation of CLL cells *in vitro* increased the expression of the anti‐apoptotic proteins Bcl‐XL, Bfl‐1, and Mcl‐1, which is consistent with the anti‐apoptotic profile of LN‐residing CLL cells *in vivo* [11, 12, 13, 14]. Previously, we conducted a comprehensive investigation of CD40‐mediated NF‐κB activation and subsequent Bcl‐XL regulation [15]. Moreover, we previously found that the T cell‐derived cytokines IL‐21 and IL‐4 differentially affect Bcl‐XL expression and sensitivity to venetoclax *in vitro* and that both are abundantly expressed in the CLL microenvironment [12, 16, 17, 18]. To further expand on this aspect, here we investigated how Bcl‐XL is regulated in the context of JAK–STAT signalling.

IL‐21 and IL‐4 are upstream activators of the JAK–STAT signalling pathway. Upon binding to their cognate receptors, receptor‐associated Janus kinases (JAKs) become activated and phosphorylated, thereby creating docking sites for signal transducers and activators of transcription (STATs) at the intracellular tail of the receptor. These events result in the phosphorylation and activation of STATs, which may translocate to the nucleus and bind DNA to directly regulate gene expression [19]. Whereas IL‐21 signals via JAK1/3 to STAT3, IL‐4 signals via JAK1/3 to STAT6 [20]. Activation of STATs is associated with the phosphorylation of specific residues in the transactivation domain at the C‐terminus, which contains a crucial tyrosine residue of which phosphorylation initiates dimerization of inactive monomers [21].

Stimulation of interleukin receptors is associated with divergent STAT activation [20], but it is unknown how IL‐21 and IL‐4 affect STAT activation in CLL and consequently influence the expression of Bcl‐XL. Bcl‐XL plays a particularly important role in shifting venetoclax sensitivity versus resistance of CLL cells in the context of CD40 stimulation [12, 15, 22]. However, direct targeting of Bcl‐XL by BH3 mimetics is associated with the induction of on‐target dose‐dependent thrombocytopenia, and thus regulators of Bcl‐XL expression might provide valuable therapeutic targets for CLL treatment [23]. Bcl‐XL is regulated by the canonical and non‐canonical NF‐κB pathways following CD40 stimulation [15], but mechanistic insight into how cytokine signals contribute to or dominate the regulation of Bcl‐XL is currently lacking and prompted us to investigate the downstream signalling pathways involved. We observed that IL‐21 and IL‐4 exert opposing effects on CD40‐mediated Bcl‐XL expression, where IL‐21/STAT3 signalling reduced and IL‐4/STAT6 signalling augmented Bcl‐XL expression via direct transcriptional regulation as well as by interfering with NF‐κB signalling activity, thereby influencing CLL drug resistance.

## 2 Materials and methods

### 2.1 Patient material

After obtaining written informed consent, patient blood was collected during diagnostic or follow‐up procedures at the Department of Hematology of the Academic Medical Center Amsterdam. This study was approved by the AMC Ethical Review Board under the number METC 2013/159 and conducted in accordance with the Declaration of Helsinki. Blood mononuclear cells of patients with CLL, obtained after Ficoll density gradient centrifugation (Pharmacia Biotech, Roosendaal, The Netherlands) were cryopreserved and stored as previously described [24]. The expression of CD5 and CD19 (Beckton Dickinson (BD) Biosciences, San Jose, CA, USA) on leukaemic cells was assessed by flow cytometry (FACScanto; BD Biosciences). The CLL samples included in this study contained 85–99% CD5^+^/CD19^+^ cells. The patient characteristics of the primary samples used in this study are depicted in Table S1.

### 2.2 Reagents

Venetoclax was purchased from Active Biochem (Bonn, Germany). A‐1331852 was purchased from Chemietek (Indianapolis, IN, USA). Ruxolitinib was purchased from Selleckchem (Houston, TX, USA). AS1517499 was purchased from Selleckchem. CW15337 was obtained from Prof. Dr. Simon Mackay (University of Strathclyde, Glasgow, UK) and previously described [15].

### 2.3 Cell culture and detection of apoptosis

Lymphocytes from CLL patients were co‐cultured with NIH3T3 fibroblasts stably transfected with human CD40L or negative control as described before [10]. After 24 h, the cells were detached and incubated with or without drugs for an additional 24 h. CLL cell viability was measured as before [13]. Specific apoptosis is defined as [% cell death in treated cells] − [% cell death in medium control]/[% viable cells medium control] × 100%.

### 2.4 Western blot analysis

Whole‐cell lysates were prepared using RIPA buffer. Subcellular fractionation was performed using the NE‐PER kit (ThermoFisher, Waltham, MA, USA). Western blot analysis was performed using standard techniques [24]. The membranes were probed with the following antibodies: anti‐Bcl‐XL, p65, p‐p65, p100/p52, p‐STAT3, p‐STAT6, Bcl‐2, TBP (Cell Signaling, Boston, MA, USA), and actin (Santa Cruz Biotechnology, Dallas, TX, USA). An Odyssey Imager (Li‐Cor Biosciences, Lincoln, NE, USA) was used as the detection method, according to the manufacturer's protocol.

### 2.5 Reverse transcription‐multiplex ligation‐dependent probe amplification assay and real‐time polymerase chain reaction

RNA was isolated using the GenElute Mammalian Total RNA Miniprep kit (Sigma‐Aldrich, Saint Louis, MO, USA), and cDNA was synthesized by reverse transcriptase reactions according to the manufacturer's instructions (Promega, Madison, WI, USA). Reverse transcription‐multiplex ligation‐dependent probe amplification assay (RT‐MLPA) procedure (MRC, Amsterdam, the Netherlands) was performed as described previously [25]. Real‐time polymerase chain reaction products were amplified in a Fast SYBR green (Life Technologies, Carlsbad, CA, USA) reaction (40 cycles of 5 s at 95 °C followed by 30 s at 60 °C) and the following primers: Bcl‐XL‐F (GTATTGGTGAGTCGGATCGC), Bcl‐XL‐R (TGCTGCATTGTTCCCATAGA), IκBα‐F (CTCCCCCTACCAGCTTACCT), IκBα‐R (TAGGGCAGCTCATCCTCTGT), HPRT‐F (CCTGGCGTCGTGATTAGTGA), and HPRT‐R (CGAGCAAGACGTTCAGTCCT).

### 2.6 Luciferase reporter gene experiments

A basic pGL3 luciferase reporter vector (Promega) was used to construct a reporter plasmid of the *Bcl‐XL* (*BCL2L1*) promoter. The primers used for the *BCL2L1* promoter were 5′‐CAGACAAAGTGCTTAACCACAAG‐3′ and 5′‐TTTTATAATAGGGATGGGCTCAACC‐3′. HEK293T cells were cotransfected with 1.5 μg of luciferase reporter plasmid and 1.5 μg empty vector, STAT3C‐GFP, or STAT6 vector (Addgene, Watertown, MA, USA). Polyethylenimine (Polysciences, Inc., Warrington, PA, USA) was used for transfection, and cells transfected with STAT6 were also stimulated with IL‐4 (25 ng·mL^−1^, Thermo Fisher Scientific, Waltham, MA, USA). After 24 h, luciferase activity was determined using a BioTek Synergy‐HT (Winooski, VT, USA).

### 2.7 DNA‐binding ELISA

Nuclear extracts of CLL cells were prepared using an NE‐PER kit (ThermoFisher). DNA binding of p65 and p52 DNA binding were determined using the TransAM NF‐κB kit (Active Motif, Carlsbad, CA, USA). STAT3 DNA binding was determined using the TransAM STAT3 kit (Active Motif).

### 2.8 Bcl‐XL promoter ELISA

Nuclear extracts of CLL cells were prepared using an NE‐PER kit (ThermoFisher). Biotinylated oligonucleotides of 20–50 bp were designed of different regions of the Bcl‐XL promoter. The oligonucleotides were coupled to their unlabeled complementary oligonucleotides to create double‐stranded oligonucleotides, which were subsequently coupled to streptavidin‐coated plates. After incubating the nuclear extracts and washing away the unbound fraction, transcription factors were detected using the following antibodies: rabbit‐anti‐p65, p100/p52 (Cell Signaling, Danvers, MA, USA), and STAT3 (Active Motif, TransAM STAT3 kit). Anti‐rabbit‐poly‐HRP (ThermoFisher) or anti‐rabbit‐HRP (Active Motif, TransAM STAT3 kit) secondary antibodies were used. Finally, after applying the 3,3′,5,5′‐tetramethylbenzidine substrate solution, the signal was detected using BioTek Synergy‐HT.

### 2.9 *In situ* proximity ligation assay

Primary CLL cells were cultured for 24 h on 3T3 or 3T40 fibroblasts, supplemented with IL‐21 or IL‐4. Subsequently, the cells were fixed, permeabilized, and attached to poly‐d‐lysine‐coated glass slides. The following primary antibodies were used: STAT3, STAT6, p‐p65, p65, and p100/52 (Cell Signaling). isPLA was performed using anti‐mouse PLUS and anti‐rabbit MINUS probes, according to the manufacturer's instructions (Merck, Darmstadt, Germany). Slides were analysed by confocal microscopy and quantification was performed using leica analysis software (Leica, Wetzlar, Germany).

### 2.10 Statistics

The student's t‐test or paired *t*‐test was used to analyse (paired) observations. The one‐way or two‐way ANOVA with multiple testing corrections was used to analyse differences between groups. *P < 0.05; **P < 0.01; ***P < 0.001; ****P < 0.0001.

## 3 Results

### 3.1 IL‐21 and IL‐4 exert opposing effects on venetoclax sensitivity via Bcl‐XL expression mediated by STAT3 and STAT6 signalling

**Fig. 1:**
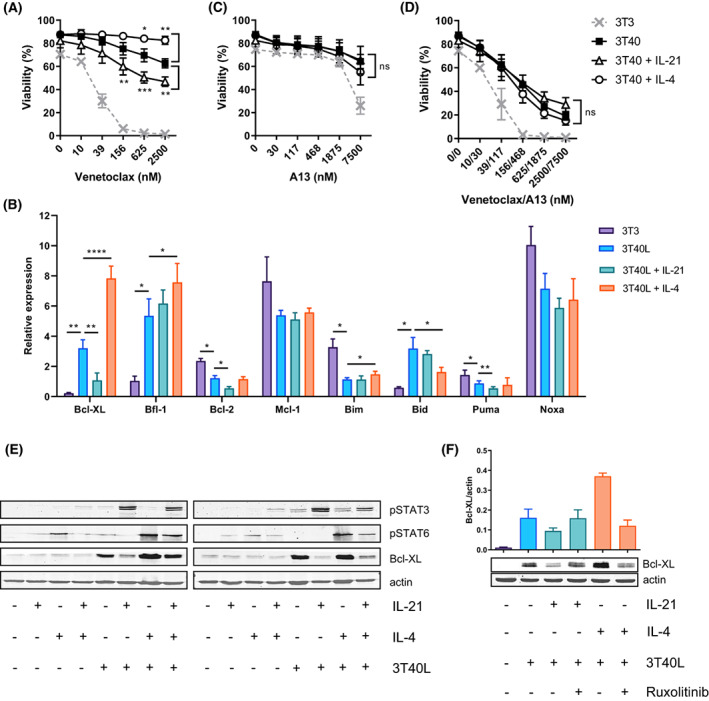
Il‐21 and IL‐4 exert opposing effects on venetoclax sensitivity via Bcl‐XL expression mediated by STAT3 and STAT6 signalling. (A) Chronic lymphocytic leukaemia (CLL) cells were cultured on 3T3 or 3T40L fibroblasts and supplemented with 25 ng·mL^−1^ IL‐21 or IL‐4 for 72 h. After detachment, cells were incubated with 0–2500 nm venetoclax for 24 h (*n* = 4). Bars represent the mean ± SEM, *P < 0.05, **P < 0.01, ***P < 0.001 (paired *t*‐test). (B) mRNA expression of Bcl‐2 family members was measured by RT‐MLPA in CLL cells 24 h after stimulation with CD40L with or without 25 ng·mL^−1^ IL‐21 or IL‐4, normalized to the sum of all data. Bars represent the mean ± SEM (*n* = 6), *P < 0.05, **P < 0.01, ****P < 0.0001 (one‐way ANOVA). (C, D) CLL cells were cultured on 3T3 or 3T40L fibroblasts and supplemented with 25 ng·mL^−1^ IL‐21 or IL‐4 for 72 h. After detachment, cells were incubated with 0–7500 nm A‐1331852 (*n* = 4) (C) or combined with 0–2500 nm venetoclax (*n* = 4) (D) for 24 h. Averaged data of 4 CLL samples are shown. Bars represent the mean ± SEM (paired *t*‐test). (E) CLL cells were cultured on 3T3 or 3T40L fibroblasts and supplemented with 25 ng·mL^−1^ IL‐21 or IL‐4 for 24 h. Protein lysates were probed for pSTAT3, pSTAT6, Bcl‐XL and Actin as loading control. Blots from 2 representative CLL samples are shown. (F) CLL cells were cultured on 3T3 or 3T40L fibroblasts and supplemented with 25 ng·mL^−1^ IL‐21 or IL‐4 and treated with 1 μm ruxolitinib for 24 h (*n* = 2). Protein lysates were probed for Bcl‐XL and Actin as loading control. Densitometric analysis shows averaged data of 2 CLL samples. Bars represent the mean ± SEM. A13, a‐1331852.

As previously described, CD40 stimulation by CD40L‐expressing fibroblasts resulted in significant venetoclax resistance of CLL cells, which we and others showed to be correlated with upregulation of the Bcl‐2 family members Bcl‐XL, Mcl‐1, and Bfl‐1 [11, 13] (Fig. 1A). Consistent with previous findings [12], IL‐21 sensitized CD40‐stimulated CLL cells to venetoclax, whereas IL‐4 further promoted venetoclax resistance. Upon screening the effects of cytokine stimulation on the expression of several Bcl‐2 family members, we found that only Bcl‐XL was differentially regulated by both IL‐21 and IL‐4 (Fig. 1B). This suggested that Bcl‐XL is the predominant factor influencing venetoclax sensitivity upon cytokine signalling. Consistently, treatment with the BH3 mimetic A‐1331852 specifically targeting Bcl‐XL, normalized the differences in venetoclax sensitivity between CD40‐activated CLL cells stimulated with or without cytokines, confirming by pharmacological means that the opposing effects of IL‐21 and IL‐4 are mediated via Bcl‐XL (Fig. 1C,D). Furthermore, we investigated individual CLL patients carrying mutations in NF‐κB‐related genes. CD40‐mediated venetoclax resistance was induced to different extents, yet a shift in sensitivity upon IL‐21 or IL‐4 stimulation was observed in all cases (Fig. S1A–H). This suggests that these distinct mutations in NF‐κB‐related pathways did not substantially affect the crosstalk with JAK–STAT signalling. Next, we investigated how IL‐21 and IL‐4 affect STAT activation in CLL and consequently influence Bcl‐XL expression. CD40‐induced Bcl‐XL expression was downregulated by IL‐21 stimulation, which was associated with pSTAT3 activation (Fig. 1E). In contrast, IL‐4 stimulation further increased CD40‐induced Bcl‐XL expression, associated with the activation of pSTAT6. To further demonstrate the essential role of STAT activation in the regulation of Bcl‐XL, the JAK1/2 inhibitor ruxolitinib was used to block the JAK–STAT signalling pathway. Inhibition by ruxolitinib abrogated the effects of IL‐21 and IL‐4 on CD40‐mediated Bcl‐XL expression, confirming that their effects on Bcl‐XL were mediated by JAK–STAT signalling (Fig. 1F). The combination of IL‐21 and IL‐4, as they are present in the microenvironment [16, 17, 18], showed differential effects on Bcl‐XL expression across patients. Therefore, single cytokine stimulations were investigated in further experiments.

### 3.2 STAT3 and STAT6 regulate Bcl‐XL expression at the transcriptional level

**Fig. 2:**
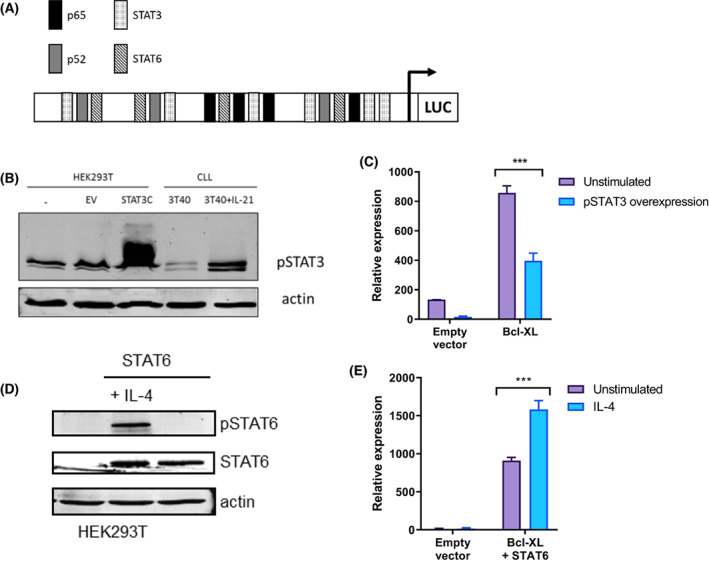
STAT3 And STAT6 regulate Bcl‐XL expression at the transcriptional level. (A) Schematic representation of the used Bcl‐XL promoter luciferase constructs. STAT3 and STAT6‐binding sites in the Bcl‐XL promoter were predicted using the JASPAR database [26]. (B) HEK293T cells were transfected with constitutive active STAT3 mutant (STAT3C) plasmid or empty vector. Chronic lymphocytic leukaemia (CLL) cells were cultured on 3T40 with IL‐21 as a positive control. Protein lysates were probed for pSTAT3 and actin as loading control. (C) HEK293T cells were transfected with Bcl‐XL promoter luciferase construct and cotransfected with STAT3C plasmid. Promoter activity was measured, and results are shown as mean ± SEM (*n* = 5). ***P < 0.001 (student's *t*‐test). (D) HEK293T cells were transfected with STAT6 plasmid and stimulated with 25 ng·mL^−1^ IL‐4. Protein lysates were probed for pSTAT6, STAT6, and actin as loading control. (E) HEK293T cells were transfected with Bcl‐XL promoter luciferase construct and STAT6 plasmid and stimulated with IL‐4. Promoter activity was measured and results are shown as mean ± SEM (*n* = 6). ***P < 0.001 (student's *t*‐test). EV, empty vector; LUC, luciferase reporter; STAT3C, constitutive active STAT3 mutant.

We next investigated how STAT signalling may regulate the transcription of Bcl‐XL. Though Bcl‐XL transcription was differentially regulated by IL‐21 and IL‐4, this was not the case for the transcription of Bfl‐1, another Bcl‐2 family member that is also regulated by NF‐κB [13, 15], indicating that the effects of IL‐21 and IL‐4 are specific for Bcl‐XL. Consistently, multiple high‐affinity STAT3 and STAT6 binding sites were predicted within the Bcl‐XL promoter region using the JASPAR database [26] (Fig. 2A). To further investigate how STAT3 may result in repression while STAT6 may result in induction of Bcl‐XL transcription, we performed reporter assays using Bcl‐XL promoter luciferase reporter constructs. As reporter assays are not possible in primary CLL cells, we investigated these aspects in HEK293T cells. Since HEK293T cells express endogenous pSTAT3 at basal levels, HEK293T cells were cotransfected with a constitutively active STAT3 mutant (STAT3C) to induce pSTAT3 overexpression (Fig. 2B). Upon introduction of pSTAT3 overexpression, Bcl‐XL promoter activity was significantly reduced (Fig. 2C). Since HEK293T cells do not express endogenous STAT6, HEK293T cells were cotransfected with STAT6 and subsequently stimulated with IL‐4 to induce STAT6 phosphorylation (Fig. 2D). Activation of STAT6 upon IL‐4 stimulation significantly increased Bcl‐XL promoter activity (Fig. 2E). These observations suggest that STAT3 negatively regulates Bcl‐XL transcription and STAT6 positively regulates it by directly binding to its promoter.

### 3.3 STAT3 and STAT6 do not disrupt the DNA‐binding activity of NF‐κB

**Fig. 3:**
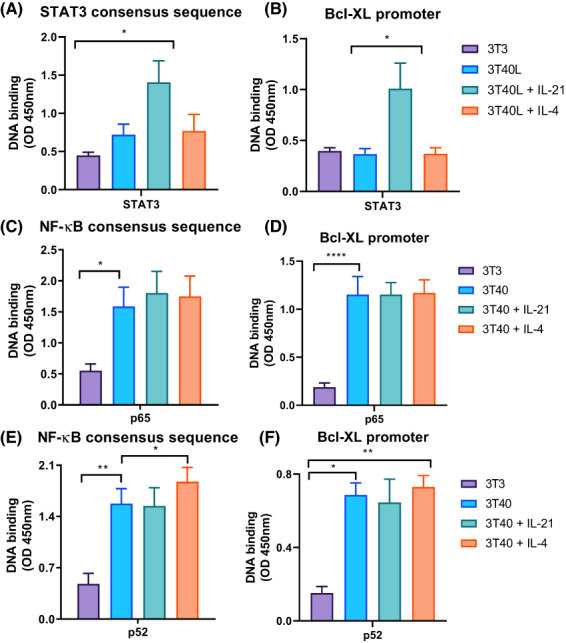
STAT3 and STAT6 do not disrupt the DNA‐binding activity of NF‐κB. Chronic lymphocytic leukemia (CLL) cells were cultured on 3T3 or 3T40L fibroblasts and supplemented with 25 ng·mL^−1^ IL‐21 or IL‐4 for 24 h. (A, B) Nuclear lysates were analysed for binding of STAT3 in a DNA‐binding ELISA using STAT3 consensus binding sites (*n* = 4) (A) or in a custom DNA‐binding ELISA using oligonucleotides containing Bcl‐XL promoter regions (*n* = 4) (B). Bars represent the mean ± SEM, *P < 0.05 (one‐way ANOVA). (C, D) Nuclear lysates were analysed for binding of p65 in a commercial DNA‐binding ELISA using NF‐κB consensus binding sites (*n* = 7) (C) or in a custom DNA‐binding ELISA using oligonucleotides containing Bcl‐XL promoter regions (*n* = 5) (D). Bars represent the mean ± SEM, *P < 0.05, ****P < 0.0001 (one‐way ANOVA). (E, F) Nuclear lysates were analysed for binding of p52 in a commercial DNA‐binding ELISA using NF‐κB consensus binding sites (*n* = 7) (E) or in a custom DNA‐binding ELISA using oligonucleotides containing Bcl‐XL promoter regions (*n* = 4) (F). Bars represent the mean ± SEM, *P < 0.05, **P < 0.01 (one‐way ANOVA). OD, optical density.

Having confirmed the direct binding of STAT3 and STAT6 to the Bcl‐XL promoter in HEK293T cells, we next aimed to test the DNA‐binding capacity of STAT3 and STAT6 in primary CLL cells. We observed a significant enrichment of STAT3 upon IL‐21 stimulation, suggesting that the DNA‐binding activity of STAT3 is dependent on active IL‐21/STAT3 signalling (Fig. 3A,B). Due to technical limitations, the DNA‐binding activity of STAT6 could not be investigated.

Although STAT3 and STAT6 affect the transcriptional regulation of Bcl‐XL, Bcl‐XL is primarily a downstream target of NF‐κB signalling. Therefore, we investigated whether JAK–STAT signalling affects the DNA‐binding capacity of NF‐κB in primary CLL cells. To this end, we measured the DNA‐binding activity of the NF‐κB regulators p65 and p52. We observed CD40‐mediated activation of canonical p65 and determined that DNA‐binding activity was unaffected by cytokine stimulation (Fig. 3C,D). While CD40‐induced DNA‐binding of non‐canonical p52 was unaffected upon IL‐21 stimulation, it increased upon IL‐4 stimulation (Fig. 3E,F). These results suggest that STAT3 and STAT6 do not disrupt the DNA‐binding capacity of NF‐κB.

### 3.4 STAT3 inhibits Bcl‐XL expression by repressing canonical NF‐κB signalling

**Fig. 4:**
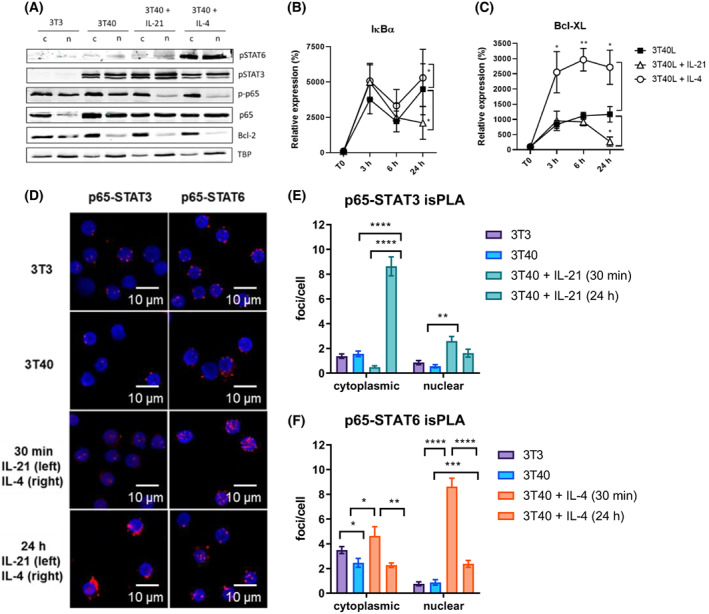
STAT3 inhibits Bcl‐XL expression by repressing canonical NF‐kB signalling. (A) Chronic lymphocytic leukemia (CLL) cells were cultured on 3T3 or 3T40L fibroblasts and supplemented with 25 ng·mL^−1^ IL‐21 or IL‐4 for 24 h. Cytoplasmic (c) and nuclear (n) lysates were probed for pSTAT6, pSTAT3, p‐p65, p65, Bcl‐2 as cytoplasmic loading control and TBP as nuclear loading control. Two patient samples were investigated, one representative example is illustrated. (B, C) mRNA expression of Bcl‐XL (B) and IκBα (C) were analysed and normalized to HPRT. Bars represent the mean ± SEM (*n* = 6), *P < 0.05, **P < 0.01 (paired *t*‐test). (D) isPLA was carried out following the manufacturer's protocol using rabbit‐anti‐p65 primary antibodies in combination with mouse‐anti‐STAT3 or mouse‐anti‐STAT6 antibodies. Three patient samples were investigated, one representative example is illustrated. Cells were analysed by confocal microscopy. Scale bars represent 10 μm as indicated. (E, F) Imaging data was quantified using leica software. Bars represent the mean ± SEM (36–64 cells analysed per condition), *P < 0.05, **P < 0.01, ***P < 0.001, ****P < 0.0001 (two‐way ANOVA). h, hours; min, minutes; T0, time point zero; TBP, TATA‐binding protein.

Next, we investigated the effects of JAK–STAT signalling on NF‐κB activation and translocation. Primary CLL cells were cultured on either 3T3 or 3T40 fibroblasts, supplemented with IL‐21 or IL‐4 for 24 h. Protein lysates of subcellular fractions were blotted with Bcl‐2 and TBP as cytoplasmic and nuclear loading controls, respectively (Fig. 4A). CD40 stimulation resulted in induction of canonical NF‐κB signalling, as observed by nuclear translocation of p65. Upon cytokine stimulation with either IL‐21 or IL‐4, total fractions of p65 did not significantly change, but nuclear p‐p65 levels disappeared. Importantly, loss of phosphorylation at this site (Ser536) is associated with a loss of transcriptional activity of p65 [27].

The most well‐known negative feedback loop of canonical NF‐κB signalling involves cytoplasmic translocation via IκB [28]. However, the transcription of IκBα was not significantly affected by cytokine stimulation during initial NF‐κB activation (up to 6 h, Fig. 4B). In contrast, Bcl‐XL transcription upon IL‐4 stimulation reached a peak at 3 h after NF‐κB activation and remained high despite the loss of p65 phosphorylation at 24 h (Fig. 4C). These observations suggest that STAT‐mediated control of Bcl‐XL transcription occurs independently of IκB. Furthermore, IL‐21 stimulation showed no effect on IκBα or Bcl‐XL transcription for up to 6 h, but showed significant inhibition of both at 24 h, suggesting attenuation of canonical NF‐κB signalling.

Next, we performed *in situ* proximity ligation assays (isPLAs) targeting NF‐κB and STAT proteins to investigate whether these proteins could interact directly and whether their subcellular localization was affected, which could lead to differences in Bcl‐XL expression. In the 3T3 and 3T40 conditions, inactive p65‐STAT complexes were observed in the cytoplasm, indicating that p65 and STAT do not require activation in order to interact (Fig. 4D–F). Upon combined CD40 and IL‐21 stimulation, p65‐STAT3 complexes accumulated in the nucleus at 30 min, whereas at 24 h barely any complexes were observed in the nucleus. This indicates that the nuclear p65‐STAT3 complexes were actively relocated to the cytoplasm. In contrast, IL‐4 stimulation resulted in significant enrichment of nuclear p65‐STAT6 complexes at both 30 min and 24 h after stimulation. Although NF‐κB and STAT may still be localized as single transcription factors (Fig. S2A,B), these observations suggest that p65‐STAT3 complexes are sequestered in the cytoplasm, whereas p65‐STAT6 complexes remain in the nucleus. Furthermore, to abrogate p65‐STAT3 cytoplasmic relocation upon IL‐21 stimulation, we used the inhibitor Selinexor to inhibit Exportin 1, which mediates STAT3 nuclear export [29]. Selinexor treatment prevented nuclear export of p65‐STAT3 complexes, yet did not rescue Bcl‐XL transcription upon IL‐21 stimulation, suggesting that although p65‐STAT3 complexes are still present in the nucleus, they are not bound to the DNA (Fig. S3A–D). In support of this, the p65‐STAT3 complexes present in the nucleus consist of unphosphorylated p65 and are thus inactive (Fig. S4). Together, these data suggest that STAT3 signalling represses canonical NF‐κB signalling via rapid relocation of p65 into the cytoplasm, thereby attenuating the transcription and expression of Bcl‐XL.

### 3.5 STAT6 increases Bcl‐XL expression by promoting non‐canonical NF‐κB signalling

**Fig. 5:**
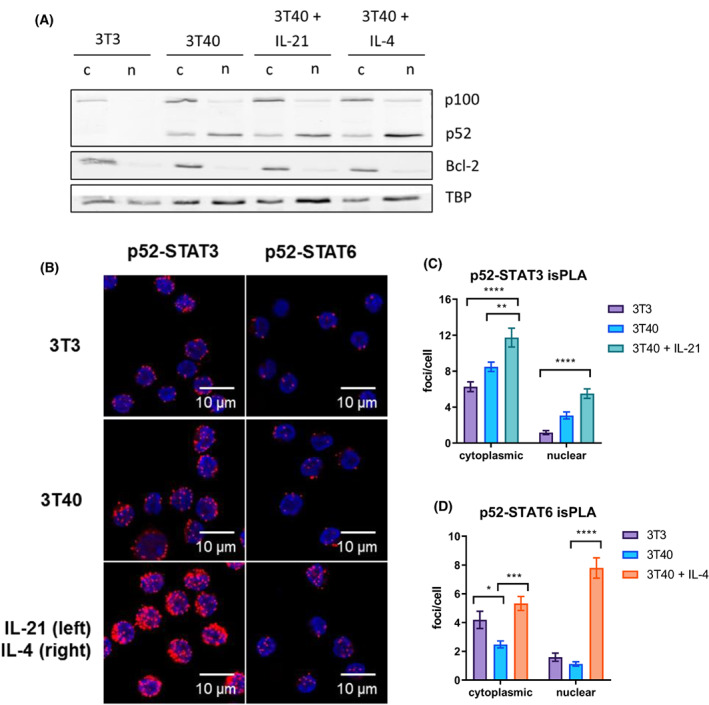
STAT6 increases Bcl‐XL expression by promoting non‐canonical NF‐κB signalling. (A) Chronic lymphocytic leukemia (CLL) cells were cultured on 3T3 or 3T40L fibroblasts and supplemented with 25 ng·mL^−1^ IL‐21 or IL‐4 for 24 h. Cytoplasmic (c) and nuclear (n) lysates were probed for p100, p52, Bcl‐2 as cytoplasmic loading control, and TBP as nuclear loading control. Two patient samples were investigated, one representative example is illustrated. (B) isPLA was carried out following the manufacturer's protocol using rabbit‐anti‐p100/p52 primary antibodies in combination with mouse‐anti‐STAT3 or mouse‐anti‐STAT6 antibodies. Three patient samples were investigated, one representative example is illustrated. Scale bars represent 10 μm as indicated. (C, D) Imaging data were quantified using leica software. Bars represent the mean ± SEM (41–78 cells analysed per condition), *P < 0.05, **P < 0.01, ***P < 0.001, ****P < 0.0001 (two‐way ANOVA). TBP, TATA‐binding protein.

Although Bcl‐XL is regulated by both canonical and non‐canonical NF‐κB pathways in CLL, the non‐canonical NF‐κB pathway is the dominant signalling route for prolonged expression of Bcl‐XL [13]. Therefore, we also investigated the effects of JAK–STAT signalling on the non‐canonical NF‐κB pathway. Consistent with the DNA‐binding assays shown in Fig. 3, CD40 and IL‐4 stimulation resulted in elevated nuclear p52 levels (Fig. 5A). In resting cells, NIK protein is continuously degraded by TRAF3 [30]. We previously showed that upon CD40 stimulation of CLL cells, NIK is stabilized [15], which is considered a key step in non‐canonical NF‐κB signalling, as it regulates the processing of the precursor p100 into p52 [31]. To extend this, isPLA was performed using an antibody targeting both p100 and p52. Resting cells in the 3T3 condition showed inactive p100‐STAT complexes in the cytoplasm (Fig. 5B–D). Upon CD40 stimulation and subsequent processing of p100 into p52, p100/p52‐STAT complexes were still observed in the cytoplasm due to inactive STAT. Stimulation with either IL‐21 or IL‐4 resulted in significant enrichment of NF‐κB‐STAT complexes in both cytoplasm and nucleus, likely representing p100‐STAT complexes in the cytoplasm and p52‐STAT complexes in the nucleus. Although upon IL‐21 stimulation more p52‐STAT3 complexes were observed in the cytoplasm than in the nucleus, there still seemed to be active non‐canonical NF‐κB signalling due to the enrichment of nuclear p52‐STAT3 complexes. These data suggest that IL‐4‐mediated STAT6 signalling promotes non‐canonical NF‐κB signalling via NIK by promoting the processing of p100 into p52, thereby upregulating the expression of Bcl‐XL.

### 3.6 Targeting NIK sensitizes resistant CLL cells to venetoclax

**Fig. 6:**
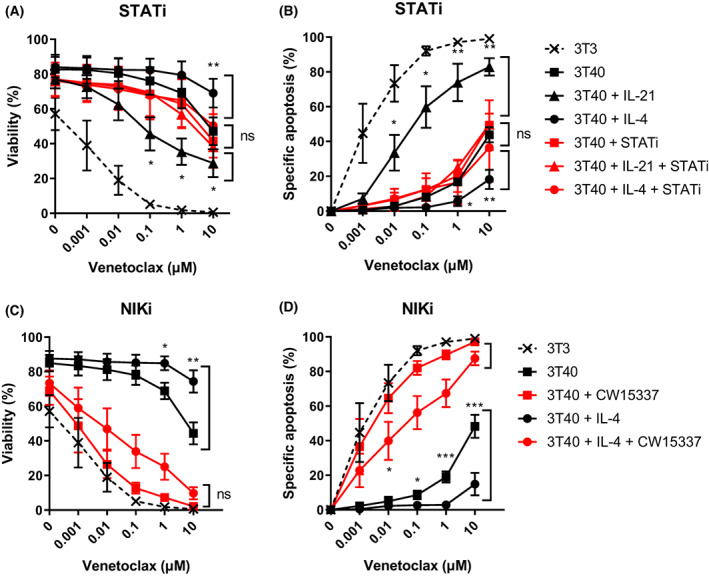
Targeting NIK sensitizes resistant CLL cells to venetoclax. (A, B) Chronic lymphocytic leukaemia (CLL) cells were cultured on 3T3 or 3T40L fibroblasts and supplemented with 25 ng·mL^−1^ IL‐4 for 24 h and/or 0.5 μm AS1517499. After detachment, cells were incubated with 0–10 μm venetoclax. Averaged data of three CLL samples are shown. Bars represent the mean ± SEM, *P < 0.05, **P < 0.01 (paired *t*‐test). (C, D) CLL cells were cultured on 3T3 or 3T40L fibroblasts and supplemented with 25 ng·mL^−1^ IL‐4 for 24 h and/or 0.5 μm CW15337. After detachment, cells were incubated with 0–10 μm venetoclax. Averaged data of six CLL samples are shown. Bars represent the mean ± SEM, *P < 0.05, **P < 0.01, ****P < 0.001 (paired *t*‐test). STATi, STAT inhibitor; NIKi, NIK inhibitor CW15337.

To link the differential effects of IL‐21 and IL‐4 to the balance between venetoclax sensitivity and resistance, we applied the STAT inhibitor AS1517499 or the NIK‐inhibitory compound CW15337, which has previously been shown to specifically inhibit non‐canonical NF‐κB signalling [15]. STAT inhibition normalized the effects of IL‐21 and IL‐4 on venetoclax resistance to the level of 3T40‐activated CLL cells (Fig. 6A,B), confirming the enhancing and attenuating effects of IL‐4 and IL‐21, respectively. Finally, we investigated whether NIK could be a potential therapeutic target to mitigate 3T40/IL‐4‐mediated resistance to venetoclax by treating 3T40/IL‐4‐stimulated CLL cells with the NIK‐inhibiting compound CW15337. NIK inhibition increased specific apoptosis from 48% to 97% in 3T40‐stimulated CLL cells, substantially abrogating CD40‐mediated venetoclax resistance (Fig. 6C,D). Even combined 3T40/IL‐4 stimulation, which provided almost absolute resistance against venetoclax with a maximum specific apoptosis of 15%, could be strongly reverted by NIK inhibition, increasing specific apoptosis to 88%. In summary, these data suggest that targeting NIK may be an effective approach to abrogate microenvironment‐induced signalling and re‐sensitize CLL cells to venetoclax.

## 4 Discussion

**Fig. 7:**
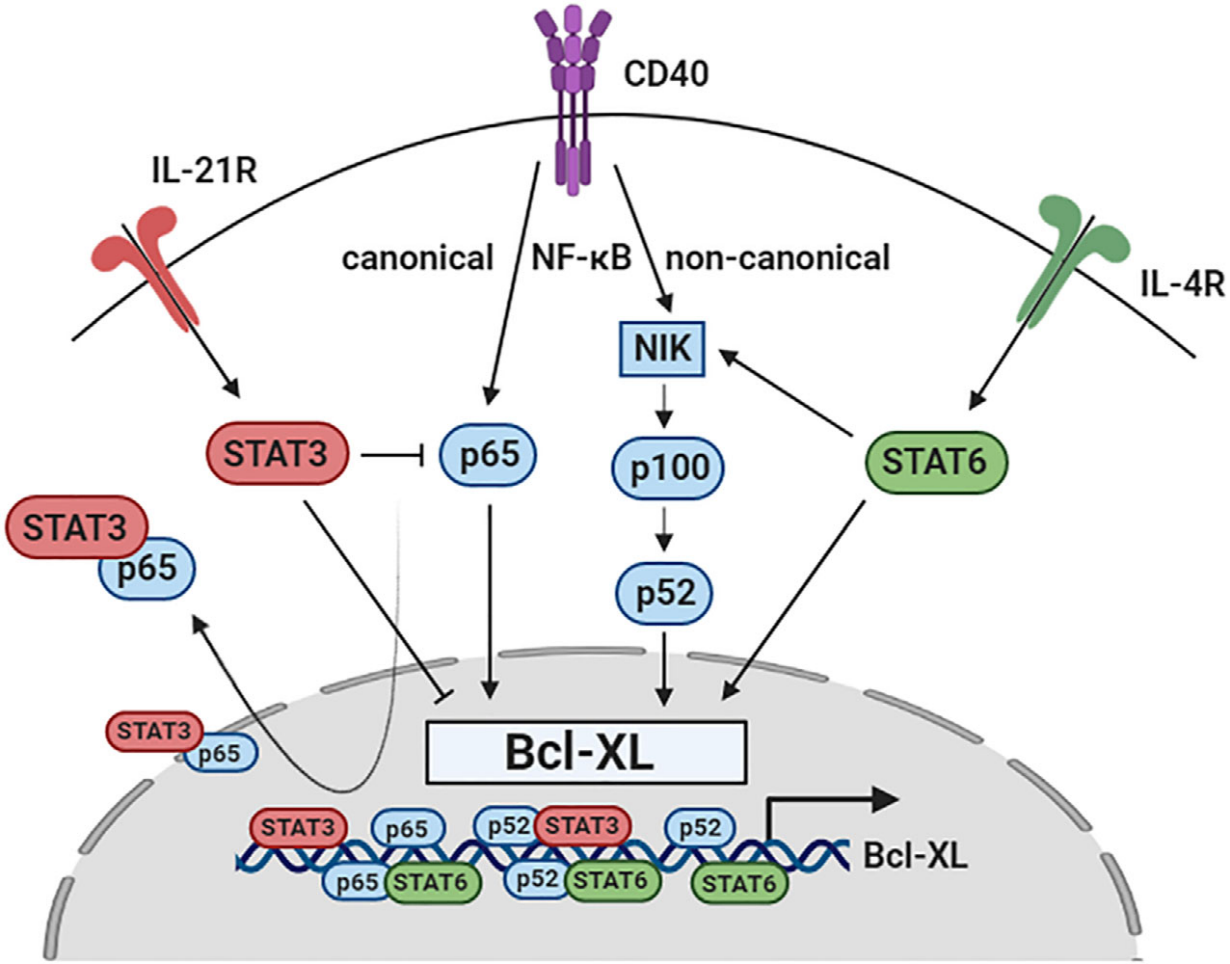
Schematic summary of results. CD40 stimulation of chronic lymphocytic leukaemia (CLL) cells leads to the upregulation of Bcl‐XL mediated by the activation of the canonical and non‐canonical NF‐κB pathways. IL‐21 stimulation inhibits Bcl‐XL expression both via direct transcriptional regulation by STAT3 as well as inhibition of p65 at the protein level via translocation and sequestering of p65 in the cytoplasm. IL‐4 stimulation further promotes the expression of Bcl‐XL via direct transcriptional regulation by STAT6 as well as promoting p52 protein levels via NIK. IL‐21R, IL‐21 receptor; IL‐4R, IL‐4 receptor.

Understanding the processes that control the upregulation of Bcl‐XL in response to microenvironmental stimuli may identify novel targets for therapy [32]. Moreover, characterization of crosstalk mechanisms with parallel signalling networks that shape the NF‐κB response may represent a clinically relevant approach [32]. Therefore, we studied the regulation of Bcl‐XL in the context of the T cell‐derived signals CD40L, IL‐21, and IL‐4. The relationship between NF‐κB and STAT3 has been repeatedly described as synergistic, where STAT3 promotes the expression of Bcl‐XL and protects cells from apoptosis [33, 34, 35]. In contrast, here, we showed a clear antagonistic role for STAT3 in the NF‐κB‐mediated expression of Bcl‐XL in primary CLL. Whereas IL‐21 stimulation reduced Bcl‐XL via transcriptional regulation by STAT3 and inhibition of canonical NF‐κB signalling, IL‐4 stimulation further increased Bcl‐XL via transcriptional regulation by STAT6 and enhanced non‐canonical NF‐κB signalling (Fig. 7).

DNA‐binding assays confirmed the direct binding of STAT3 to the Bcl‐XL promoter in primary CLL cells and showed that JAK–STAT signalling did not compete or interfere with the binding of NF‐κB to the Bcl‐XL promoter. Next, we investigated the effects of JAK–STAT signalling on NF‐κB activation and signalling activity. Cytokine stimulation with either IL‐21 or IL‐4 resulted in a loss of Ser536 phosphorylation of nuclear p65, which is associated with a loss of p65 transcriptional activity without affecting DNA‐binding activity [36, 37, 38, 39], consistent with our data. Nuclear translocation of p‐p65(Ser536) has previously been shown to be independent of IκB and without interaction with cofactors [27]. Therefore, loss of p65 phosphorylation upon cytokine stimulation may allow interaction with cofactors such as STATs, which may subsequently influence NF‐κB activity and transcription of Bcl‐XL and other target genes. Notably, isPLA experiments targeting p‐p65(Ser536) did not show any nuclear complexes with STAT3 or STAT6 upon NF‐κB/JAK–STAT activation, indicating that all the nuclear p65‐STAT6 complexes we observed consisted of unphosphorylated p65 (Fig. S4). Although loss of p65 transcriptional activity fits with STAT3‐mediated inhibition of Bcl‐XL, it does not correspond with STAT6‐mediated upregulation of Bcl‐XL. A possible explanation could be that transactivation of STAT6 upon interaction with p65 is sufficient to rescue the loss of p65 transcriptional activity [40]. Consistently, p65‐STAT6 complexes were enriched in the nucleus upon IL‐4 stimulation, suggesting that STAT6 may induce target gene expression as a complex with p65.

We showed that the interaction of STAT3 and STAT6 with NF‐κB may affect their subcellular localization, revealing an additional layer of complexity in STAT‐NF‐κB signalling crosstalk, which may function as a mechanism to shape the NF‐κB response. Time course experiments showed an early increase of Bcl‐XL transcription upon IL‐4 stimulation, whereas IL‐21‐mediated inhibition of Bcl‐XL was observed at later time points, possibly due to the accumulation of cytoplasmic STAT3 levels [41]. Since CD40 stimulation of CLL cells results in low‐level pSTAT3 activation independent of cytokine stimulation [18, 42], it is likely that STAT3 may act as a negative regulator to dampen or fine‐tune the NF‐κB response. Though loss of p65 phosphorylation would enable negative feedback by IκB, transcriptional time course experiments suggested that STAT3‐mediated inhibition of canonical NF‐κB signalling was independent of IκB negative feedback. This is consistent with the enrichment of cytoplasmic STAT3‐p65 complexes and a previous study showing that STAT3 may bind p65 in competition with IκB [43]. Several studies have reported NF‐κB activation upon STAT3 inhibition based on the nuclear translocation of p65, indeed suggesting that STAT3 sequesters p65 in the cytoplasm [44, 45]. Other studies confirmed the binding of STAT3‐p65 transcription factors to inhibit the transcription of certain NF‐κB target genes [43], but did not show binding of this complex to the Bcl‐XL promoter [46].

When checking for non‐canonical NF‐κB signalling, we observed increased nuclear p52 protein levels upon IL‐4 stimulation, which was consistent with increased p52 DNA binding. These data suggest enhanced processing of p100 to p52 via NIK, thereby representing an additional mechanism for JAK–STAT signalling to shape the NF‐κB response and influence the expression of Bcl‐XL. It was previously shown that IL‐4 may induce p100 processing into p52, specifically in healthy B cells [47], and our data suggest that this is also the case for CLL cells.

In summary, we have demonstrated multiple regulatory layers involving stimulus‐dependent effects that influence NF‐κB activity to regulate the target gene Bcl‐XL. A subsequent goal is to take advantage of the accumulated knowledge of the mechanisms used to confer NF‐κB selectivity to develop therapeutic strategies in order to modulate Bcl‐XL‐mediated drug resistance in CLL. We applied an inhibitor of NIK, which largely normalized the differences in venetoclax resistance caused by IL‐4 stimulation, suggesting that targeting NF‐κB signalling also eliminates NF‐κB/STAT signalling crosstalk. An alternative approach could be to address JAK–STAT signalling, of which some strategies have been previously investigated. IL‐21 has previously been shown to mediate pro‐apoptotic effects in several CLL studies [48, 49, 50]. Notably, recombinant IL‐21 was tested in combination with rituximab in a phase I clinical trial involving CLL patients [51]. Combination therapy with IL‐21 was well‐tolerated and responses were typically longer than the patient's previous response to rituximab‐based therapy. There have been no follow‐up studies concerning IL‐21‐based therapy in CLL, but the development of novel antibody‐cytokine fusions that deliver cytokines to specific cells may be applicable [52]. However, a risk to consider is that IL‐21 promotes antigen‐independent proliferation of CLL cells *in vitro* [18, 42]. In addition, various inhibitory strategies against JAK–STAT signalling are being pursued [21]. Some studies have focused on inhibiting STAT dimerization using peptides or small‐molecule inhibitors targeting the tyrosine kinases that phosphorylate STAT monomers [53, 54]. The JAK1/2 inhibitor ruxolitinib showed promising results in clinical trials and caused recompartmentalization of CLL cells from tissues to peripheral blood, which may provide a rationale for combination therapy with venetoclax [55], as currently explored in the context of AML (NCT03874052). Additionally, JAK–STAT inhibition may be favorable for clinical application as opposed to cytokine therapy, as JAK inhibitors also block cytokine‐induced proliferation of CLL cells [54]. Alternative approaches include the use of oligonucleotides that function as decoy STAT DNA‐binding sites to compete with DNA‐binding to endogenous promoter sequences [56].

## 5 Conclusions

In conclusion, we demonstrated that JAK–STAT signalling may shape the NF‐κB response in CLL towards venetoclax sensitivity or resistance via Bcl‐XL, involving multiple regulatory crosstalk mechanisms, thereby providing multiple potential targets for the downregulation of Bcl‐XL, which could be applied in combination therapy for CLL.

## Conflict of interest

The authors declare no conflict of interest.

## Author contributions

MVH performed the research, analysed data, and wrote the paper. RT, DB, and DB performed the research and analysed data. FdB provided patient samples. SM provided vital reagents and served as scientific advisor. JD and CM provided patient samples. AK provided patient samples and wrote the manuscript. EE designed the study and wrote the manuscript.

### Peer review

The peer review history for this article is available at https://publons.com/publon/10.1002/1878‐0261.13364.

## Supporting information


**Fig. S1.** A‐H) Chronic lymphocytic leukemia (CLL) cells were cultured on 3T3 or 3T40L fibroblasts and supplemented with 25ng/mL IL‐21 or IL‐4 for 72 hours. After detachment, cells were incubated with 0‐10.000nM venetoclax for 24 hours. Viability and specific apoptosis are plotted of 3 NOTCH1‐mutated patients (A‐B), 1 MYD88‐mutated patient (C‐D), 1 BIRC3‐mutated patient (E‐F) and 1 NFKBIE‐mutated patient (G‐H). Bars represent the mean ± SEM, *p<0.05, **p<0.01, ***p<0.001, ****p<0.0001 (paired t‐test). I) CLL cells were cultured on 3T3 or 3T40L fibroblasts and supplemented with 25ng/mL IL‐21 or IL‐4 for 24 hours. Protein lysates were probed for Bcl‐XL and actin as loading control.

**Fig. S2.** A) Chronic lymphocytic leukemia (CLL) cells were cultured on 3T3 or 3T40L fibroblasts and supplemented with 25ng/mL IL‐21 for 24 hours. Cells were stained for DAPI (blue), STAT3 (green) and p65 (red) and imaged by confocal microscopy. Scale bars represent 10 μm as indicated. B) CLL cells were cultured on 3T3 or 3T40L fibroblasts and supplemented with 25ng/mL IL‐4 for 24 hours. Cells were stained for DAPI (blue), STAT6 (green) and p65 (red) and imaged by confocal microscopy. Scale bars represent 10 μm as indicated.

**Fig. S3.** A) Chronic lymphocytic leukemia (CLL) cells were cultured on 3T3 or 3T40L fibroblasts and supplemented with 25ng/mL IL‐21 or IL‐4 for 24 hours while simultaneously treated with a titration of Selinexor. B) mRNA expression of Bcl‐XL was analyzed and normalized to HPRT. Bars represent the mean ± SEM (n=3). C) isPLA was carried out following the manufacturer's protocol using rabbit‐anti‐p65 primary antibodies in combination with mouse‐anti‐STAT3 antibodies. Scale bars represent 10 μm as indicated. D) Imaging data was quantified using LEICA software. Bars represent the mean ± SEM, ***p<0.001, ****p<0.0001 (two‐way ANOVA).

**Fig. S4.** Chronic lymphocytic leukemia (CLL) cells were cultured on 3T3 or 3T40L fibroblasts and supplemented with 25ng/mL IL‐21 or IL‐4 for 24 hours. isPLA was carried out following the manufacturer's protocol using rabbit‐anti‐p‐p65 primary antibodies in combination with mouse‐anti‐STAT3 or mouse‐anti‐STAT6 antibodies. Scale bars represent 10 μm as indicated.

**Table S1.** Clinical characteristics of the patient samples used in this study.

## Data Availability

All data included in the manuscript is available on request from the corresponding author.

## References

[1] Fischer K , Al‐Sawaf O , Bahlo J , Fink AM , Tandon M , Dixon M , et al. Venetoclax and Obinutuzumab in patients with CLL and coexisting conditions. N Engl J Med. 2019;380:2225–36. 10.1056/NEJMoa1815281 31166681

[2] Roberts AW , Davids MS , Pagel JM , Kahl BS , Puvvada SD , Gerecitano JF , et al. Targeting BCL2 with Venetoclax in relapsed chronic lymphocytic leukemia. N Engl J Med. 2016;374:311–22. 10.1056/NEJMoa1513257 26639348 PMC7107002

[3] Seymour JF , Kipps TJ , Eichhorst B , Hillmen P , D'Rozario J , Assouline S , et al. Venetoclax‐rituximab in relapsed or refractory chronic lymphocytic leukemia. N Engl J Med. 2018;378:1107–20. 10.1056/NEJMoa1713976 29562156

[4] Blombery P , Anderson MA , Gong JN , Thijssen R , Birkinshaw RW , Thompson ER , et al. Acquisition of the recurrent Gly101Val mutation in BCL2 confers resistance to Venetoclax in patients with progressive chronic lymphocytic leukemia. Cancer Discov. 2019;9:342–53. 10.1158/2159-8290.CD-18-1119 30514704

[5] Blombery P , Thompson ER , Nguyen T , Birkinshaw RW , Gong JN , Chen X , et al. Multiple BCL2 mutations cooccurring with Gly101Val emerge in chronic lymphocytic leukemia progression on venetoclax. Blood. 2020;135:773–7. 10.1182/blood.2019004205 31951646 PMC7146015

[6] Burger JA , Gandhi V . The lymphatic tissue microenvironments in chronic lymphocytic leukemia: in vitro models and the significance of CD40‐CD154 interactions. Blood. 2009;114:2560–1; author reply 2561–2562. 10.1182/blood-2009-06-228981 19762501 PMC4969051

[7] Burger JA , Tsukada N , Burger M , Zvaifler NJ , Dell'Aquila M , Kipps TJ . Blood‐derived nurse‐like cells protect chronic lymphocytic leukemia B cells from spontaneous apoptosis through stromal cell‐derived factor‐1. Blood. 2000;96:2655–63. 11023495

[8] Ghia P , Strola G , Granziero L , Geuna M , Guida G , Sallusto F , et al. Chronic lymphocytic leukemia B cells are endowed with the capacity to attract CD4+, CD40L+ T cells by producing CCL22. Eur J Immunol. 2002;32:1403–13. 10.1002/1521-4141(200205)32:5&lt;1403::AID-IMMU1403&gt;3.0.CO;2-Y 11981828

[9] Kurtova AV , Balakrishnan K , Chen R , Ding W , Schnabl S , Quiroga MP , et al. Diverse marrow stromal cells protect CLL cells from spontaneous and drug‐induced apoptosis: development of a reliable and reproducible system to assess stromal cell adhesion‐mediated drug resistance. Blood. 2009;114:4441–50. 10.1182/blood-2009-07-233718 19762485 PMC4081374

[10] Panayiotidis P , Jones D , Ganeshaguru K , Foroni L , Hoffbrand AV . Human bone marrow stromal cells prevent apoptosis and support the survival of chronic lymphocytic leukaemia cells in vitro. Br J Haematol. 1996;92:97–103. 8562418 10.1046/j.1365-2141.1996.00305.x

[11] Smit LA , Hallaert DY , Spijker R , de Goeij B , Jaspers A , Kater AP , et al. Differential Noxa/Mcl‐1 balance in peripheral versus lymph node chronic lymphocytic leukemia cells correlates with survival capacity. Blood. 2007;109:1660–8. 10.1182/blood-2006-05-021683 17038534

[12] Thijssen R , Slinger E , Weller K , Geest CR , Beaumont T , van Oers MH , et al. Resistance to ABT‐199 induced by microenvironmental signals in chronic lymphocytic leukemia can be counteracted by CD20 antibodies or kinase inhibitors. Haematologica. 2015;100:e302–6. 10.3324/haematol.2015.124560 25957396 PMC5004430

[13] Tromp JM , Tonino SH , Elias JA , Jaspers A , Luijks DM , Kater AP , et al. Dichotomy in NF‐kappaB signaling and chemoresistance in immunoglobulin variable heavy‐chain‐mutated versus unmutated CLL cells upon CD40/TLR9 triggering. Oncogene. 2010;29:5071–82. 10.1038/onc.2010.248 20581863

[14] Vogler M , Butterworth M , Majid A , Walewska RJ , Sun XM , Dyer MJ , et al. Concurrent up‐regulation of BCL‐XL and BCL2A1 induces approximately 1000‐fold resistance to ABT‐737 in chronic lymphocytic leukemia. Blood. 2009;113:4403–13. 10.1182/blood-2008-08-173310 19008458

[15] Haselager M , Thijssen R , West C , Young L , Van Kampen R , Willmore E , et al. Regulation of Bcl‐XL by non‐canonical NF‐kappaB in the context of CD40‐induced drug resistance in CLL. Cell Death Differ. 2021;28:1658–68. 10.1038/s41418-020-00692-w 33495554 PMC8167103

[16] Aguilar‐Hernandez MM , Blunt MD , Dobson R , Yeomans A , Thirdborough S , Larrayoz M , et al. IL‐4 enhances expression and function of surface IgM in CLL cells. Blood. 2016;127:3015–25. 10.1182/blood-2015-11-682906 27002119

[17] Ahearne MJ , Willimott S , Pinon L , Kennedy DB , Miall F , Dyer MJ , et al. Enhancement of CD154/IL4 proliferation by the T follicular helper (Tfh) cytokine, IL21 and increased numbers of circulating cells resembling Tfh cells in chronic lymphocytic leukaemia. Br J Haematol. 2013;162:360–70. 10.1111/bjh.12401 23710828

[18] Pascutti MF , Jak M , Tromp JM , Derks IA , Remmerswaal EB , Thijssen R , et al. IL‐21 and CD40L signals from autologous T cells can induce antigen‐independent proliferation of CLL cells. Blood. 2013;122:3010–9. 10.1182/blood-2012-11-467670 24014238

[19] O'Shea JJ , Schwartz DM , Villarino AV , Gadina M , McInnes IB , Laurence A . The JAK‐STAT pathway: impact on human disease and therapeutic intervention. Annu Rev Med. 2015;66:311–28. 10.1146/annurev-med-051113-024537 25587654 PMC5634336

[20] Murray PJ . The JAK‐STAT signaling pathway: input and output integration. J Immunol. 2007;178:2623–9. 10.4049/jimmunol.178.5.2623 17312100

[21] Miklossy G , Hilliard TS , Turkson J . Therapeutic modulators of STAT signalling for human diseases. Nat Rev Drug Discov. 2013;12:611–29. 10.1038/nrd4088 23903221 PMC4038293

[22] Haselager MV , Kielbassa K , Ter Burg J , Bax DJC , Fernandes SM , Borst J , et al. Changes in Bcl‐2 members in response to ibrutinib or venetoclax uncover functional hierarchy in determining resistance to venetoclax in CLL. Blood. 2020;136:2918–26. 10.1182/blood.2019004326 32603412

[23] Tolcher AW , LoRusso P , Arzt J , Busman TA , Lian G , Rudersdorf NS , et al. Safety, efficacy, and pharmacokinetics of navitoclax (ABT‐263) in combination with irinotecan: results of an open‐label, phase 1 study. Cancer Chemother Pharmacol. 2015;76:1041–9. 10.1007/s00280-015-2882-9 26429709

[24] Hallaert DY , Jaspers A , van Noesel CJ , van Oers MH , Kater AP , Eldering E . c‐Abl kinase inhibitors overcome CD40‐mediated drug resistance in CLL: implications for therapeutic targeting of chemoresistant niches. Blood. 2008;112:5141–9. 10.1182/blood-2008-03-146704 18796631

[25] Eldering E , Spek CA , Aberson HL , Grummels A , Derks IA , de Vos AF , et al. Expression profiling via novel multiplex assay allows rapid assessment of gene regulation in defined signalling pathways. Nucleic Acids Res. 2003;31:e153. 14627843 10.1093/nar/gng153 PMC290288

[26] Mathelier A , Fornes O , Arenillas DJ , Chen CY , Denay G , Lee J , et al. JASPAR 2016: a major expansion and update of the open‐access database of transcription factor binding profiles. Nucleic Acids Res. 2016;44:D110–5. 10.1093/nar/gkv1176 26531826 PMC4702842

[27] Sasaki CY , Barberi TJ , Ghosh P , Longo DL . Phosphorylation of RelA/p65 on serine 536 defines an I{kappa}B{alpha}‐independent NF‐{kappa}B pathway. J Biol Chem. 2005;280:34538–47. 10.1074/jbc.M504943200 16105840

[28] Kearns JD , Basak S , Werner SL , Huang CS , Hoffmann A . IkappaBepsilon provides negative feedback to control NF‐kappaB oscillations, signaling dynamics, and inflammatory gene expression. J Cell Biol. 2006;173:659–64. 10.1083/jcb.200510155 16735576 PMC2063883

[29] Bhattacharya S , Schindler C . Regulation of Stat3 nuclear export. J Clin Invest. 2003;111:553–9. 10.1172/JCI15372 12588893 PMC151917

[30] Liao G , Zhang M , Harhaj EW , Sun SC . Regulation of the NF‐kappaB‐inducing kinase by tumor necrosis factor receptor‐associated factor 3‐induced degradation. J Biol Chem. 2004;279:26243–50. 10.1074/jbc.M403286200 15084608

[31] Xiao G , Harhaj EW , Sun SC . NF‐kappaB‐inducing kinase regulates the processing of NF‐kappaB2 p100. Mol Cell. 2001;7:401–9. 11239468 10.1016/s1097-2765(01)00187-3

[32] Oeckinghaus A , Hayden MS , Ghosh S . Crosstalk in NF‐kappaB signaling pathways. Nat Immunol. 2011;12:695–708. 10.1038/ni.2065 21772278

[33] Lee TL , Yeh J , Friedman J , Yan B , Yang X , Yeh NT , et al. A signal network involving coactivated NF‐kappaB and STAT3 and altered p53 modulates BAX/BCL‐XL expression and promotes cell survival of head and neck squamous cell carcinomas. Int J Cancer. 2008;122:1987–98. 10.1002/ijc.23324 18172861

[34] Li P , Harris D , Liu Z , Rozovski U , Ferrajoli A , Wang Y , et al. STAT3‐activated GM‐CSFRalpha translocates to the nucleus and protects CLL cells from apoptosis. Mol Cancer Res. 2014;12:1267–82. 10.1158/1541-7786.MCR-13-0652-T 24836891 PMC4163508

[35] Liu FT , Jia L , Wang P , Wang H , Farren TW , Agrawal SG . STAT3 and NF‐kappaB cooperatively control in vitro spontaneous apoptosis and poor chemo‐responsiveness in patients with chronic lymphocytic leukemia. Oncotarget. 2016;7:32031–45. 10.18632/oncotarget.8672 27074565 PMC5077994

[36] Haller D , Russo MP , Sartor RB , Jobin C . IKK beta and phosphatidylinositol 3‐kinase/Akt participate in non‐pathogenic gram‐negative enteric bacteria‐induced RelA phosphorylation and NF‐kappa B activation in both primary and intestinal epithelial cell lines. J Biol Chem. 2002;277:38168–78. 10.1074/jbc.M205737200 12140289

[37] Jiang X , Takahashi N , Matsui N , Tetsuka T , Okamoto T . The NF‐kappa B activation in lymphotoxin beta receptor signaling depends on the phosphorylation of p65 at serine 536. J Biol Chem. 2003;278:919–26. 10.1074/jbc.M208696200 12419817

[38] O'Mahony AM , Montano M , Van Beneden K , Chen LF , Greene WC . Human T‐cell lymphotropic virus type 1 tax induction of biologically active NF‐kappaB requires IkappaB kinase‐1‐mediated phosphorylation of RelA/p65. J Biol Chem. 2004;279:18137–45. 10.1074/jbc.M401397200 14963024

[39] Yang F , Tang E , Guan K , Wang CY . IKK beta plays an essential role in the phosphorylation of RelA/p65 on serine 536 induced by lipopolysaccharide. J Immunol. 2003;170:5630–5. 10.4049/jimmunol.170.11.5630 12759443

[40] Shen CH , Stavnezer J . Interaction of stat6 and NF‐kappaB: direct association and synergistic activation of interleukin‐4‐induced transcription. Mol Cell Biol. 1998;18:3395–404. 10.1128/mcb.18.6.3395 9584180 PMC108921

[41] Ichiba M , Nakajima K , Yamanaka Y , Kiuchi N , Hirano T . Autoregulation of the Stat3 gene through cooperation with a cAMP‐responsive element‐binding protein. J Biol Chem. 1998;273:6132–8. 10.1074/jbc.273.11.6132 9497331

[42] Slinger E , Thijssen R , Kater AP , Eldering E . Targeting antigen‐independent proliferation in chronic lymphocytic leukemia through differential kinase inhibition. Leukemia. 2017;31:2601–7. 10.1038/leu.2017.129 28462919

[43] Yang J , Liao X , Agarwal MK , Barnes L , Auron PE , Stark GR . Unphosphorylated STAT3 accumulates in response to IL‐6 and activates transcription by binding to NFkappaB. Genes Dev. 2007;21:1396–408. 10.1101/gad.1553707 17510282 PMC1877751

[44] McFarland BC , Gray GK , Nozell SE , Hong SW , Benveniste EN . Activation of the NF‐kappaB pathway by the STAT3 inhibitor JSI‐124 in human glioblastoma cells. Mol Cancer Res. 2013;11:494–505. 10.1158/1541-7786.MCR-12-0528 23386688 PMC3656973

[45] Nefedova Y , Cheng P , Gilkes D , Blaskovich M , Beg AA , Sebti SM , et al. Activation of dendritic cells via inhibition of Jak2/STAT3 signaling. J Immunol. 2005;175:4338–46. 10.4049/jimmunol.175.7.4338 16177074 PMC1351251

[46] Yoshida Y , Kumar A , Koyama Y , Peng H , Arman A , Boch JA , et al. Interleukin 1 activates STAT3/nuclear factor‐kappaB cross‐talk via a unique TRAF6‐ and p65‐dependent mechanism. J Biol Chem. 2004;279:1768–76. 10.1074/jbc.M311498200 14593105

[47] Thieu VT , Nguyen ET , McCarthy BP , Bruns HA , Kapur R , Chang CH , et al. IL‐4‐stimulated NF‐kappaB activity is required for Stat6 DNA binding. J Leukoc Biol. 2007;82:370–9. 10.1189/jlb.1106707 17513694

[48] de Totero D , Meazza R , Zupo S , Cutrona G , Matis S , Colombo M , et al. Interleukin‐21 receptor (IL‐21R) is up‐regulated by CD40 triggering and mediates proapoptotic signals in chronic lymphocytic leukemia B cells. Blood. 2006;107:3708–15. 10.1182/blood-2005-09-3535 16391014

[49] Gowda A , Roda J , Hussain SR , Ramanunni A , Joshi T , Schmidt S , et al. IL‐21 mediates apoptosis through up‐regulation of the BH3 family member BIM and enhances both direct and antibody‐dependent cellular cytotoxicity in primary chronic lymphocytic leukemia cells in vitro. Blood. 2008;111:4723–30. 10.1182/blood-2007-07-099531 18182577 PMC2343602

[50] Jahrsdorfer B , Blackwell SE , Wooldridge JE , Huang J , Andreski MW , Jacobus LS , et al. B‐chronic lymphocytic leukemia cells and other B cells can produce granzyme B and gain cytotoxic potential after interleukin‐21‐based activation. Blood. 2006;108:2712–9. 10.1182/blood-2006-03-014001 16809616 PMC1895576

[51] Timmerman JM , Byrd JC , Andorsky DJ , Yamada RE , Kramer J , Muthusamy N , et al. A phase I dose‐finding trial of recombinant interleukin‐21 and rituximab in relapsed and refractory low grade B‐cell lymphoproliferative disorders. Clin Cancer Res. 2012;18:5752–60. 10.1158/1078-0432.CCR-12-0456 22893631 PMC5027960

[52] Neri D . Antibody‐cytokine fusions: versatile products for the modulation of anticancer immunity. Cancer Immunol Res. 2019;7:348–54. 10.1158/2326-6066.CIR-18-0622 30824549 PMC6994246

[53] Auzenne EJ , Klostergaard J , Mandal PK , Liao WS , Lu Z , Gao F , et al. A phosphopeptide mimetic prodrug targeting the SH2 domain of Stat3 inhibits tumor growth and angiogenesis. J Exp Ther Oncol. 2012;10:155–62. 23350355 PMC4033579

[54] Hofland T , de Weerdt I , Ter Burg H , de Boer R , Tannheimer S , Tonino SH , et al. Dissection of the effects of JAK and BTK inhibitors on the functionality of healthy and malignant lymphocytes. J Immunol. 2019;203:2100–9. 10.4049/jimmunol.1900321 31511358

[55] Spaner DE , Wang G , McCaw L , Li Y , Disperati P , Cussen MA , et al. Activity of the Janus kinase inhibitor ruxolitinib in chronic lymphocytic leukemia: results of a phase II trial. Haematologica. 2016;101:e192–5. 10.3324/haematol.2015.135418 26819050 PMC5004376

[56] Sen M , Thomas SM , Kim S , Yeh JI , Ferris RL , Johnson JT , et al. First‐in‐human trial of a STAT3 decoy oligonucleotide in head and neck tumors: implications for cancer therapy. Cancer Discov. 2012;2:694–705. 10.1158/2159-8290.CD-12-0191 22719020 PMC3668699

