# Supplementary figures and images for: JAK–STAT signalling shapes the NF‐κB response in CLL towards venetoclax sensitivity or resistance via Bcl‐XL
Source: Mol Oncol. 2023 Feb 13;17(6):1112–28. doi: 10.1002/1878-0261.13364 (PMC10257415; doi:10.1002/1878-0261.13364)

## Slide 1
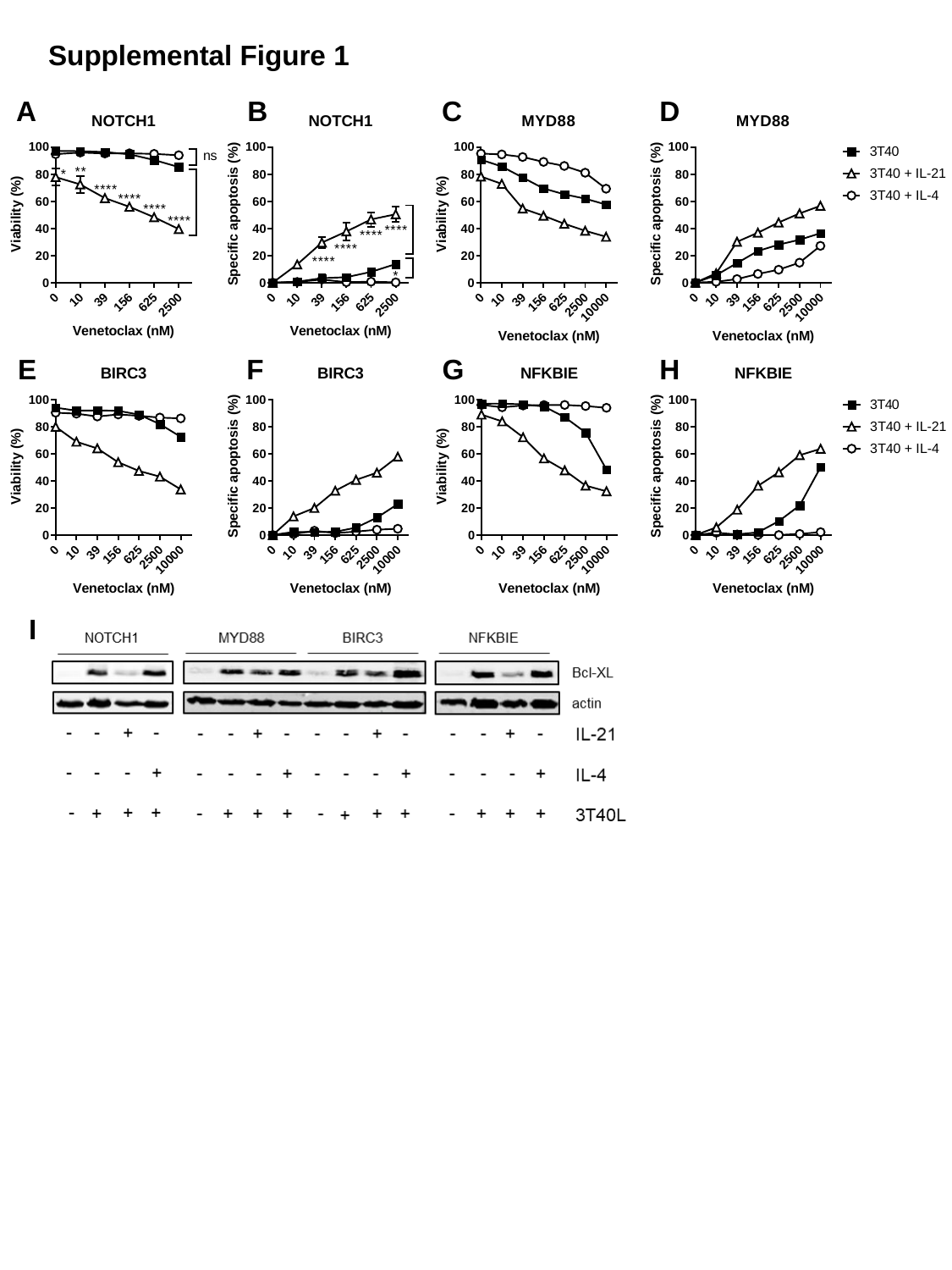

Supplemental Figure 1
A
B
C
D
E
F
G
H
I

Supplement: Supplementary file 1 — Fig. S1. A‐H) Chronic lymphocytic leukemia (CLL) cells were cultured on 3T3 or 3T40L fibroblasts and supplemented with 25ng/mL IL‐21 or IL‐4 for 72 hours. After detachment, cells were incubated with 0‐10.000nM venetoclax for 24 hours. Viability and specific apoptosis are plotted of 3 NOTCH1‐mutated patients (A‐B), 1 MYD88‐mutated patient (C‐D), 1 BIRC3‐mutated patient (E‐F) and 1 NFKBIE‐mutated patient (G‐H). Bars represent the mean ± SEM, *p<0.05, **p<0.01, ***p<0.001, ****p<0.0001 (paired t‐test). I) CLL cells were cultured on 3T3 or 3T40L fibroblasts and supplemented with 25ng/mL IL‐21 or IL‐4 for 24 hours. Protein lysates were probed for Bcl‐XL and actin as loading control. [file MOL2-17-1112-s004.ppt]

## Slide 1
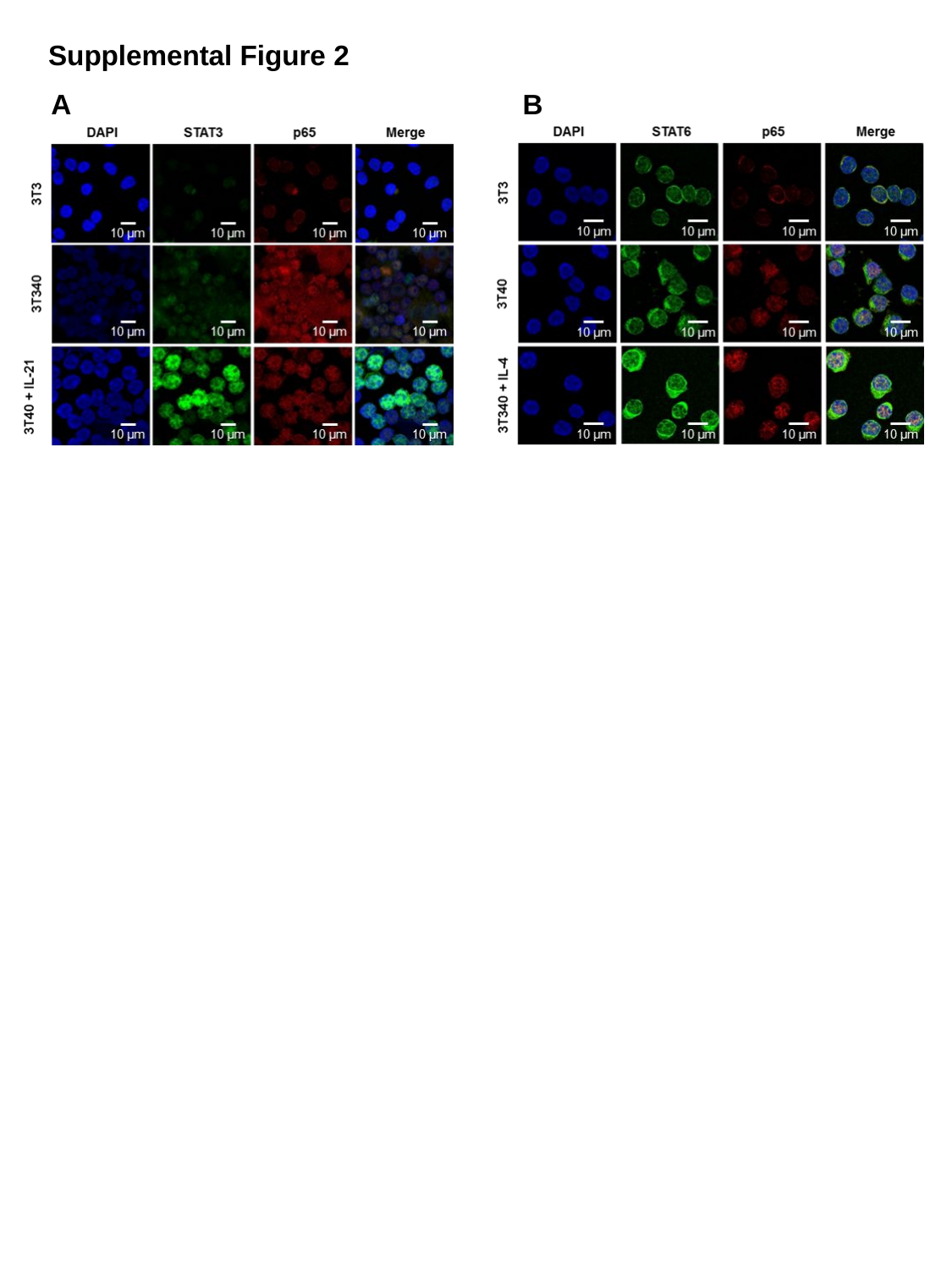

Supplemental Figure 2
A
B

Supplement: Supplementary file 2 — Fig. S2. A) Chronic lymphocytic leukemia (CLL) cells were cultured on 3T3 or 3T40L fibroblasts and supplemented with 25ng/mL IL‐21 for 24 hours. Cells were stained for DAPI (blue), STAT3 (green) and p65 (red) and imaged by confocal microscopy. Scale bars represent 10 μm as indicated. B) CLL cells were cultured on 3T3 or 3T40L fibroblasts and supplemented with 25ng/mL IL‐4 for 24 hours. Cells were stained for DAPI (blue), STAT6 (green) and p65 (red) and imaged by confocal microscopy. Scale bars represent 10 μm as indicated. [file MOL2-17-1112-s005.ppt]

## Slide 1
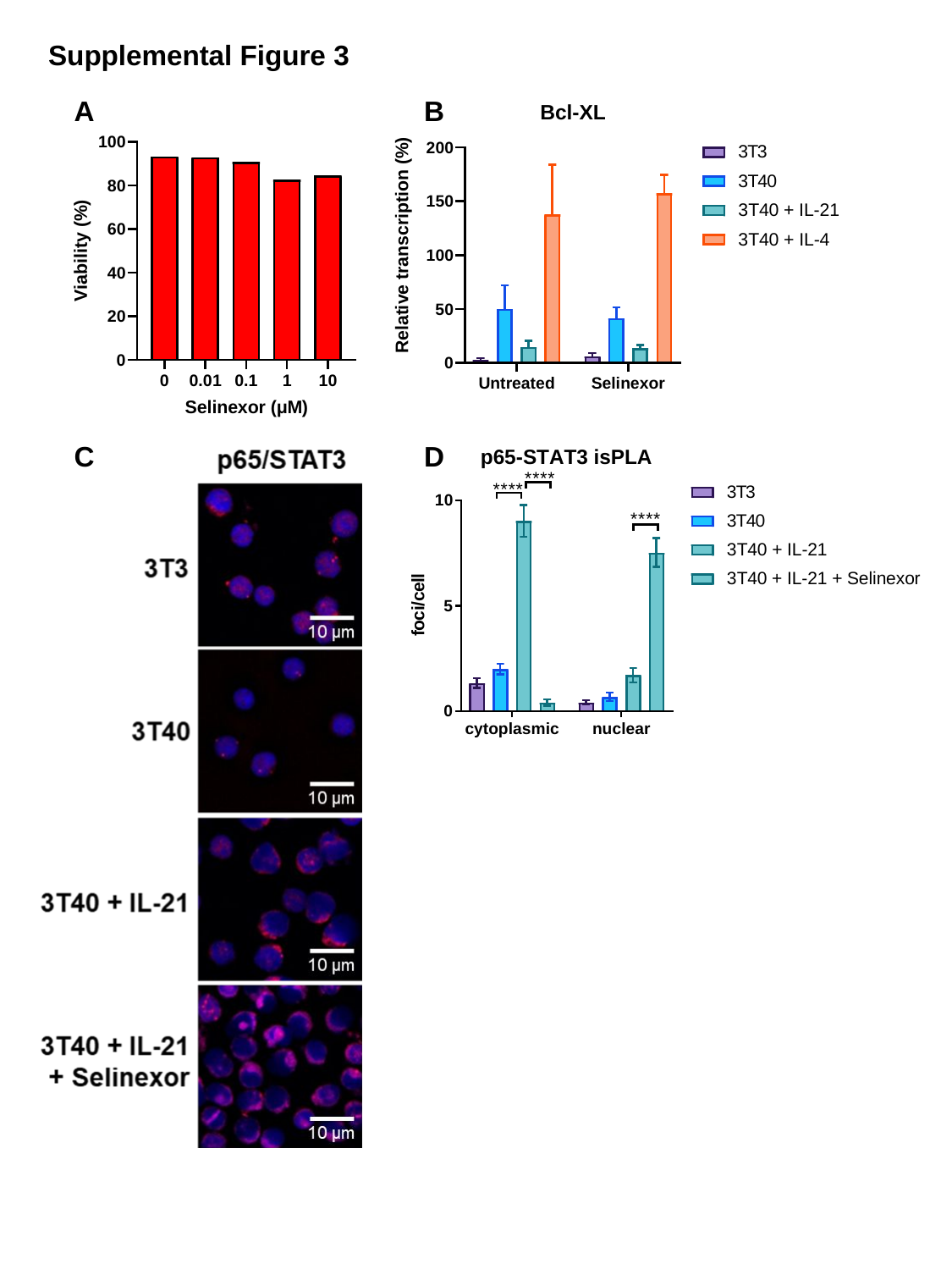

Supplemental Figure 3
A
B
C
D

Supplement: Supplementary file 3 — Fig. S3. A) Chronic lymphocytic leukemia (CLL) cells were cultured on 3T3 or 3T40L fibroblasts and supplemented with 25ng/mL IL‐21 or IL‐4 for 24 hours while simultaneously treated with a titration of Selinexor. B) mRNA expression of Bcl‐XL was analyzed and normalized to HPRT. Bars represent the mean ± SEM (n=3). C) isPLA was carried out following the manufacturer's protocol using rabbit‐anti‐p65 primary antibodies in combination with mouse‐anti‐STAT3 antibodies. Scale bars represent 10 μm as indicated. D) Imaging data was quantified using LEICA software. Bars represent the mean ± SEM, ***p<0.001, ****p<0.0001 (two‐way ANOVA). [file MOL2-17-1112-s002.ppt]

## Slide 1
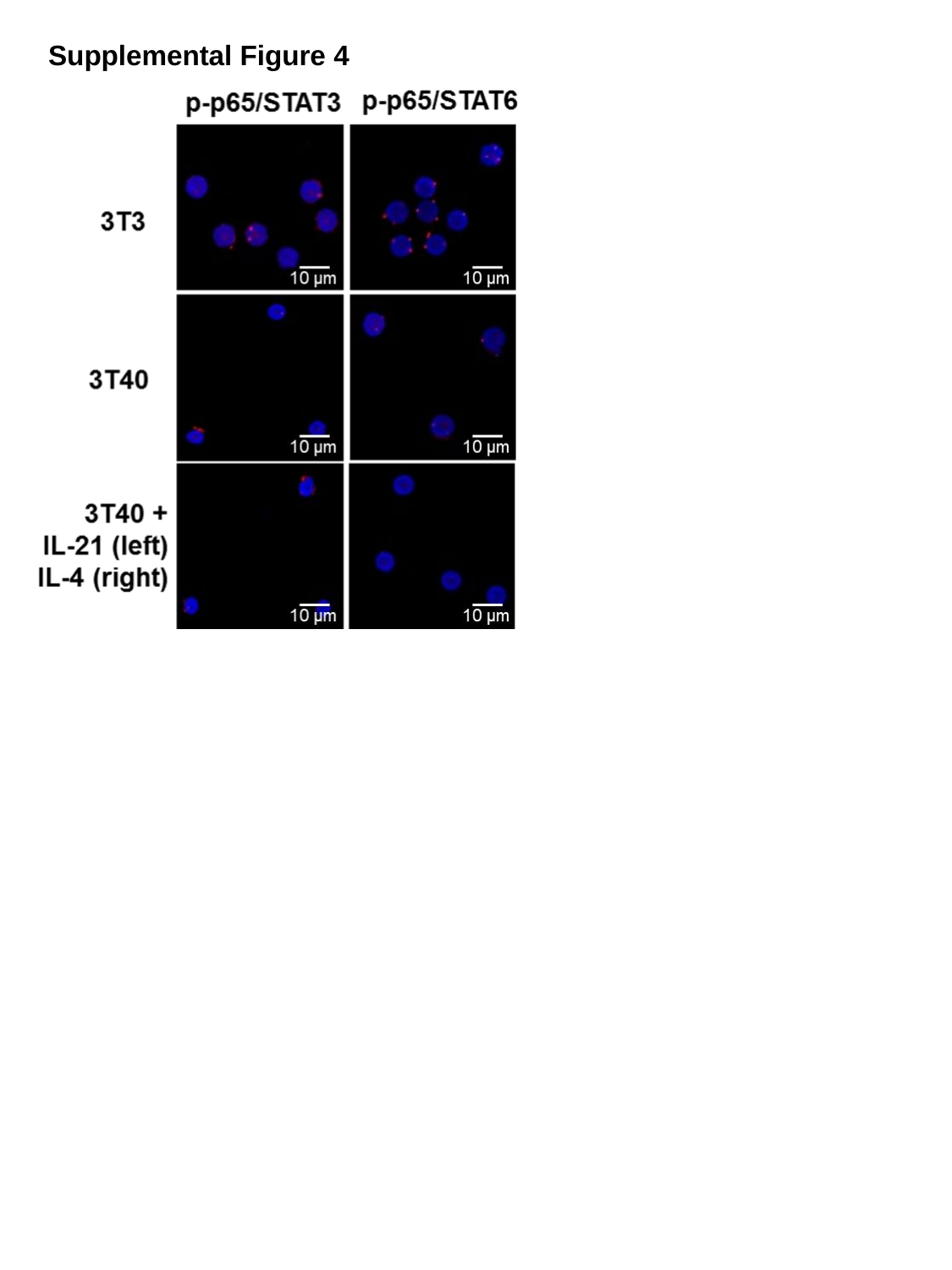

Supplemental Figure 4

Supplement: Supplementary file 4 — Fig. S4. Chronic lymphocytic leukemia (CLL) cells were cultured on 3T3 or 3T40L fibroblasts and supplemented with 25ng/mL IL‐21 or IL‐4 for 24 hours. isPLA was carried out following the manufacturer's protocol using rabbit‐anti‐p‐p65 primary antibodies in combination with mouse‐anti‐STAT3 or mouse‐anti‐STAT6 antibodies. Scale bars represent 10 μm as indicated. [file MOL2-17-1112-s001.ppt]

## Slide 1
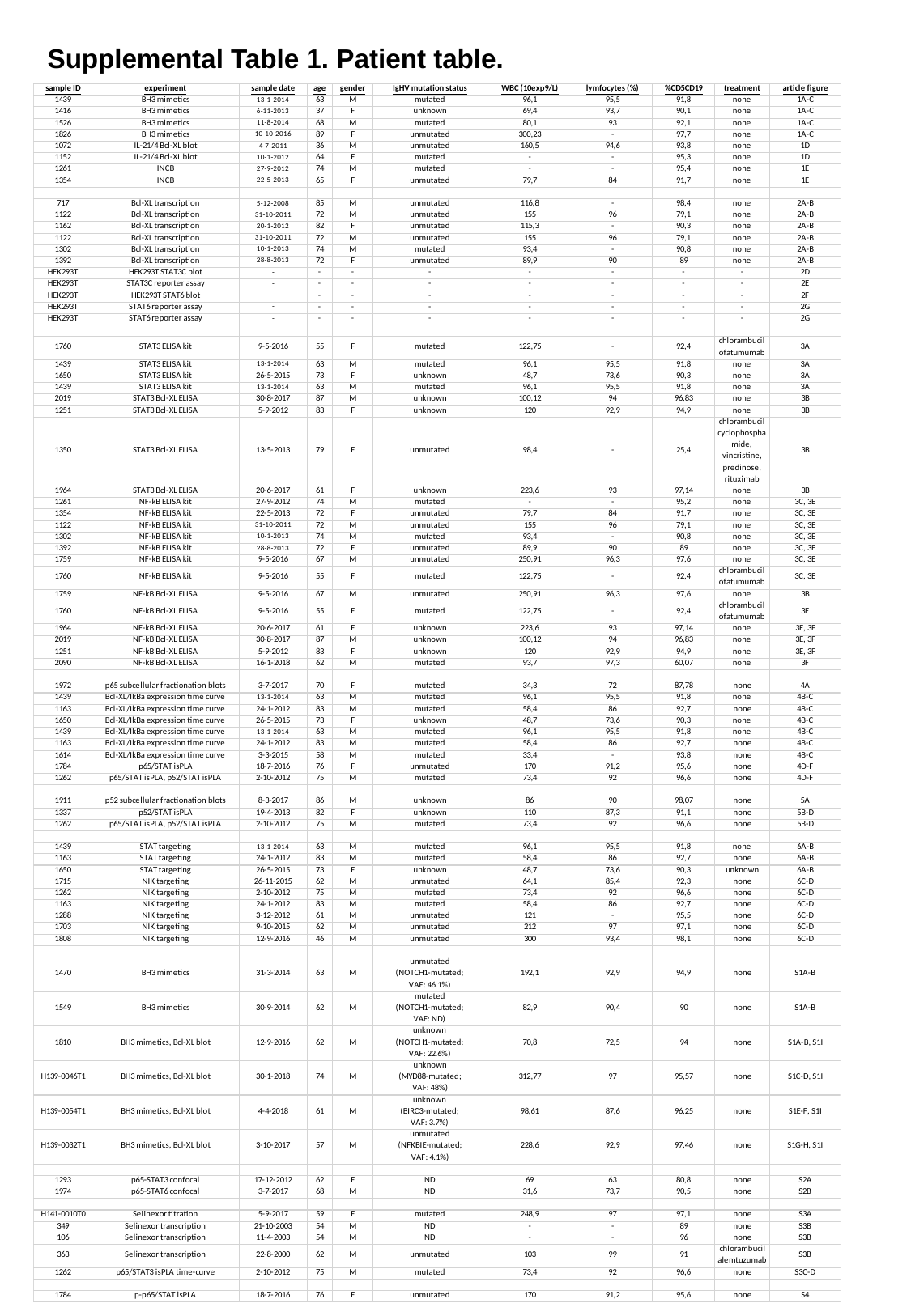

Supplemental Table 1. Patient table.

Supplement: Supplementary file 5 — Table S1. Clinical characteristics of the patient samples used in this study. [file MOL2-17-1112-s003.pptx]
